# Computer-Aided Drug Discovery Identifies Alkaloid Inhibitors of Parkinson's Disease Associated Protein, Prolyl Oligopeptidase

**DOI:** 10.1155/2021/6687572

**Published:** 2021-04-08

**Authors:** Apoorva M. Kulkarni, Shailima Rampogu, Keun Woo Lee

**Affiliations:** Division of Life Sciences, Division of Applied Life Science (BK21 Plus), Plant Molecular Biology and Biotechnology Research Center (PMBBRC), Research Institute of Natural Science (RINS), Gyeongsang National University (GNU), 501 Jinju-daero, Jinju 52828, Republic of Korea

## Abstract

Parkinson's disease is a common neurodegenerative disorder marked by the accumulation of the protein alpha synuclein. Studies have indicated the role of prolyl oligopeptidase (POP), a serine protease, in alpha synuclein accumulation. Therefore, POP emerges as an attractive medicinal target. Traditionally, most of the early medicines have been plant-based owing to their ready availability and negligible side effects. Alkaloids owing to their neurotransmitter modulatory, anti-amyloid, anti-oxidant, and anti-inflammatory activities have shown potential in neurodegenerative disease. In this work, we computationally evaluated alkaloid class of phytochemicals for their therapeutic efficacy against POP. Alkaloids were retrieved from the publically available database, Chemical Entities of Biological Interest (ChEBI), and screened for their drug likeness (Lipinski's rule of 5) and absorption, distribution, metabolism, and excretion, and toxicity (ADMET) in Discovery Studio by ensuring parameters suitable for a central nervous system disease such as blood-brain barrier (BBB) level set to ≤2, absorption level set to 0 and solubility level permitted set to 2, 3, or 4. Next, molecular docking was performed to learn about the affinity of the filtered alkaloids with the POP. Subsequently, molecular dynamic simulations were conducted to assess the reliability and stability of the alkaloid-protein complex. Our study identified metergoline, pipercallosine, celacinnine, lobeline, cystodytin G, lycoperine A, hookerianamide J, and martefragin A as putative lead compounds against POP. Among these, metergoline, pipercallosine, hookerianamide J, and lobeline showed the most promising results. These compounds demonstrated better or equivalent molecular docking scores in comparison to three POP inhibitors that had reached clinical trials, i.e., Z-321, S-17092, and JTP-4819. MD simulations indicated that these compounds remained intact at the active site while adhering to the binding mode and interaction patterns as that of the reported inhibitors. The research conducted here, therefore, provides evidence for conducting in vitro POP inhibitory studies of these newly identified plant-based POP inhibitors.

## 1. Introduction

Parkinson's disease (PD) is a chronic and progressive neurodegenerative central nervous system (CNS) disorder. Affected individuals experience difficulty in walking, tremors, stiffness in limbs, or impaired balance (https://www.ninds.nih.gov/disorders/patient-caregiver-education/hope-through-research/parkinsons-disease-hope-through-research, last accessed December 20, 2019). Largely an age-related disease, PD can be either sporadic or genetic. It is characterized by the presence of Lewy bodies which are clusters of alpha synuclein and other proteins [1]. In 2008, research done by Brandt et al. indicated that prolyl oligopeptidase (POP) stimulates the aggregation of alpha synuclein [2]. Also, Hannula and colleagues identified that POP generally co-localizes with alpha synuclein and this interaction is stronger in PD brains [3]. Therefore, POP emerges as an attractive pharmacological target.

POP (also known as PREP or prolyl endopeptidase; EC 3.4.21.26) is a large 80 KDa intracellular enzyme belonging to serine protease family. It is capable of cleaving peptides shorter than 30 amino acids after a proline residue [4]. POP is majorly expressed in brain areas populated with neuropeptide receptors [5] and influences CNS-related activities like memory, learning, and mood responses [6, 7].

POP in entirety assumes a cylindrical shape and is comprised of two domains: peptidase or catalytic domain (residues 1–72 and 428–710) containing the alpha/beta hydrolase fold, and a 7-bladed *β*-propeller domain (residues 73–427) containing seven-fold repeat of four stranded antiparallel *β* sheets. The catalytic triad consisting of Ser554, His680, and Asp641 is found at the interface of these domains. Several sub-sites form part of active site: the S1 specificity pocket comprises Phe476, Asn555, Val580, Trp595, Tyr599, and Val644 residues thus forming a hydrophobic environment for the ease of accommodating the proline or aromatic rings present in inhibitors, the less specific S2 pocket containing the guanidium ring of Arg643, and the hydrophobic S3 pocket comprising non-polar residues such as Phe173, Met235, Cys255, Ile591, and Ala594. Hydrogen bonds interactions of the residues Trp595 and Arg643 with the POP inhibitors have been shown to be of vital importance [8].

Although several research groups have focused on POP inhibition [9–11], three inhibitors Z-321, S-17092, and JTP-4819 were considered for clinical trials [12]. Crossing of blood brain barrier (BBB) poses a major concern for POP inhibitors [13]. Besides this, safety is a perennial question in drug discovery projects. In this regard, plant-based therapeutics are alluring as they have been used long in history without any adverse side effects. Berbirine and baicalein were identified as natural product inhibitors of POP with IC50 values of 145 ± 19 *μ*M and 12 ± 3 *μ*M [14, 15]. Cyclotide psysol 2 isolated from *Psychotria solitudinum* was found to inhibit POP [16]. Despite the identification of these natural product POP inhibitors, no advancement has been done and there are currently no clinically approved POP inhibitors in the market. This urges for identification of safe and novel therapeutics effective against POP. There have been reports on activity of various alkaloids in PD and other neurodegenerative diseases [17–19]. Therefore, here we have computationally evaluated alkaloids to screen for the best prospective drug candidate against POP and elucidate their mechanism of action.

## 2. Materials and Methods

In brief, structures of the alkaloids and target protein (POP) were retrieved. Alkaloids were screened for their drug likeness and ADMET properties, following which molecular docking was performed in order to access their binding to POP. Lastly, molecular dynamics simulations were conducted to evaluate the stability of the prospective drug candidates when bound to POP.

### 2.1. Structure Retrieval and Preparation of Target Protein

Structure of POP bearing PDB id: 3DDU [20] was downloaded from Protein Data Bank (PDB). Using Discovery studio v18 (DS) [21], the protein structure was cleaned by deleting heteroatoms and water molecules. Hydrogen atoms were added. Missing loops were added based on the sequence obtained from Uniprot identifier P48147 [22].

### 2.2. Alkaloids Retrieval, Drug Likeness, and ADMET Prediction

The list of alkaloid compounds was downloaded from ChEBI (Chemical Entities of Biological Interest) (https://www.ebi.ac.uk/chebi/init.do) by using the search term “alkaloid.” Retrieved compounds were first subjected to absorption, distribution, metabolism, and excretion, and toxicity (ADMET) studies. For this, DS's ADMET tool was used with a value of 0 as cutoff for ADMET_ABSORPTION_LEVEL and a value of 2, 3, or 4 as cutoff for ADMET_SOLUBILITY_LEVEL and a cutoff value of 0, 1, or 2 was used for ADMET_BBB_LEVEL. Compounds that passed ADMET were subjected to “Filter by Lipinski and Veber Rules” using the default parameters. After this, the alkaloids were minimized using “full minimization tool” in DS for carrying out the molecular docking studies with the target protein.

### 2.3. Molecular Docking

To predict the binding affinity of the filtered alkaloids with POP, molecular docking was performed using Genetic Optimisation for Ligand Docking (GOLD) version 5.2.2 [23]. The ligand binding co-ordinates were calculated by inbound co-crystal (GSK552) using “Define and edit binding site” module of DS. GoldScore fitness was used as the default scoring function. GoldScore fitness comprises hydrogen bond and van der Waals energy of protein ligand, ligand internal van der Waals energy, and ligand torsional strain energy. The best docked pose of the alkaloid with the protein was selected based on the following: it should be part of the largest cluster that obeys the co-crystal's binding mode, should have a better GoldScore fitness value than reference compounds, and should demonstrate interactions with key residues in the active site. In order to validate our docking parameters, GSK552 from PDB structure was docked and root mean square deviation (RMSD) was checked for.

### 2.4. Molecular Dynamics Simulations

To determine the reliability and consistency of the binding of the prospective drug candidates to the target protein, molecular dynamics (MD) simulations were performed using GROningen MAchine for Chemical Simulations (GROMACS) v5.0.6 package [24] and using CHARMm27 [25] as desired force field. Ligand topology was generated using SwissParam [26]. Dodecahedron system solvated with TIP3P water model was utilized for solvation. Na^+^ ions were added to neutralize the negative charge of the system. Steepest Descent was used for energy minimization. Equilibration of the system was carried out in two phases: constant number N, volume V, and temperature T (NVT) and constant number N, pressure P, and temperature T (NPT). NVT ensemble was performed at 300 K for 1 ns and NPT ensemble at 1 bar pressure for 1 ns with a Parrinello–Rahman barostat [27]. Once the system was well equilibrated, MD was carried out for 50 ns (metergoline) or 20 ns (other identified alkaloid inhibitors). MD analysis was performed using Visual Molecular Dynamics (VMD) and DS.

## 3. Results

### 3.1. Drug Likeness and ADMET

ChEBI search resulted in the retrieval of 565 alkaloid compounds (accessed on 14th August 2019). To ensure a good pharmacokinetic profile of the potential drug candidates, ADMET tests were carried out in DS. By setting 0 as cutoff value for ADMET_ABSORPTION_LEVEL, it was ensured that potential inhibitors had a good intestinal absorption. Filtering of compounds where ADMET_SOLUBILITY_LEVEL was either 2, 3, or 4 ensured that the potential inhibitors were neither too soluble nor insoluble in water. The most important factor in identifying drug for neurological diseases is their ability to penetrate blood brain barrier. Usage of 0, 1, or 2 as cutoff for ADMET_BBB_LEVEL ensured only the compounds that could pass blood brain barrier would be retained. 190 of the initial 565 compounds passed the ADMET test. Drug likeness of these 190 compounds was accessed using “Filter by Lipinski and Veber Rules” with default parameters. Lipinski's rule states that a drug-like molecule should not have more than 5 hydrogen bond donors and 10 hydrogen bond acceptors, molecular weight should not exceed 500 daltons, and LogP should not be more than 5 [28]. 189 compounds that passed drug-likeness criteria were retained and were subjected to further study.

### 3.2. Molecular Docking to Access Binding Affinity with POP

In order to determine whether the candidate inhibitors bind to POP, molecular docking was performed by employing Z-321and S-17092 as reference 1 (Ref1) and reference 2 (Ref2), respectively.

RMSD measures the average distance between the atoms of superimposed proteins and is used to measure the quality of reproduction of a known binding pose. Therefore, the lower the RMSD, the lesser the deviation from the known binding pose. The docked pose of co-crystal, i.e., GSK552 with POP resulted in an RMSD of 0.094 nm. The low RMSD ensured the credibility of our docking protocol. Using the affirmed docking parameters of GSK552, 189 candidate inhibitors were docked at the active site of POP. From the largest cluster obeying the binding mode of the co-crystal, best pose was selected based on higher score than that of reference compounds and interaction with key residues. GoldScore fitness for Ref1 and Ref2 was 70.5265 and 68.4808, respectively. Metergoline with a GoldScore fitness of 72.6547 was the only compound to score better than both Ref1 and Ref2. Pipercallosine with a GoldScore fitness of 68.6628 attained a slightly higher docking score than that of Ref2 (68.4808). Additionally, since JTP-4819 was also considered in clinical trials as POP inhibitor, we also docked it against POP. The GoldScore fitness value for JTP-4819 against POP was 60.0431. Apart from metergoline and pipercallosine, six other alkaloids, celacinnine, lobeline, cystodytin G, lycoperine A, hookerianamide J, and martefragin A, fared better than JTP-4819. These collectively will be referred to as hit candidates from now on. The docking scores of all these compounds are tabulated in Supplementary Table 1.

**Figure 1:**
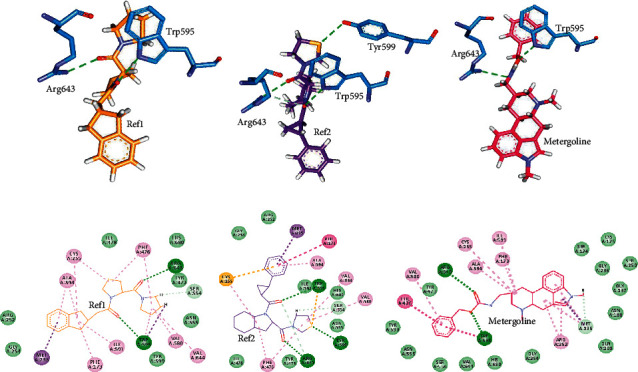
Molecular docking based intermolecular interactions. Upper panel (a–c) demonstrates hydrogen bond interactions of POP residues (blue) with (a) Ref1, (b) Ref2, and (c) metergoline. Lower panel (d–f) is the 2D representation of all the molecular interactions between POP and (d) Ref1, (e) Ref2, and (f) metergoline. Green dashed lines in upper and lower panel represent hydrogen bond. All the other dashed lines represent various types of *π* bonds. Light green colored spheres indicate the residues participating in van der Waals interactions.

After gaining knowledge on binding affinity via docking scores, we explored the interaction pattern of the hit candidates and reference compounds with POP. Because of its high docking score, we were mainly interested in learning about metergoline's interaction with POP active site residues. “Show 2d diagram” of DS was utilized to inspect the molecular interactions with POP. Ref1, Ref2, and metergoline all interacted with the active site residues. Ref1 formed hydrogen bond with Trp595 and Arg643 and Ref2 formed hydrogen bond with Trp595, Tyr599, and Arg643. Metergoline like Ref1 and Ref2 was involved in hydrogen bond formation with Arg643 and Trp595, thus maintaining the necessary hydrogen bond interactions. These hydrogen bond interactions are depicted in Figures 1(a)–1(c). Ref1 and Ref2 demonstrated non-covalent *π* interactions with Phe173, Met235, Cys255, Phe476, Val580, Ala594, Trp595, and Val644 with Ref1 showing an additional interaction with Ile591. Metergoline formed *π* interactions with Phe173, Met235, Arg252, Cys255, Phe476, Val580, Ile591, Ala594, and Trp595. van der Waals interactions were formed by both Ref1 and Ref2 with Arg252, Gly254, Tyr473, Ile478, Asn555, and His680 residues of POP. Ref1 and Ref2 developed an additional van der Waals interaction with Tyr599 and Ile591, respectively. Both Ref1 and Ref2 displayed a carbon-hydrogen bond with Ser554. Additionally, Ref2 formed a carbon-hydrogen bond with Arg643 also. Metergoline established van der Waals bonds with Ser174, Cys175, Asn188, Gln208, Gly236, Gly237, Ser250, Gly254, Tyr473, Ser554, Asn555, Tyr599, Val644, and His680 residues. Metergoline also formed a carbon-hydrogen bond with Met235. All these interactions are presented in Figures 1(d)–1(f).

We also performed interaction analysis of JTP-4819 and other hit compounds (pipercallosine, celacinnine, lobeline, cystodytin G, lycoperine A, hookerianamide J, and martefragin A) with POP. JTP-4819 demonstrated four hydrogen bonds: one each with Arg128 and Cys255 and two with Arg643. *π* interactions were seen between Phe173, Phe476, Ile591, Ala594, Trp595, and JTP-4819. With the other active site residues such as Met235, Asn555, Val580, Tyr599, and Val644, JTP-4819 established van der Waals interactions. Pipercallosine established one hydrogen bond with Arg643. Cystodytin G demonstrated a total of three hydrogen bonds, two with Cys255 and one with Tyr473. Lobeline, lycoperine A, and hookerianamide J established two hydrogen bonds. Lobeline and hookerianamide J established hydrogen bonds, one each with Cys255 and Arg643, whereas lycoperine A established hydrogen bonds, one each with Cys255 and Tyr473. Interestingly, martefragin A demonstrated 5 hydrogen bonds: a bifurcated hydrogen bond with Ser554 and His680 and one each with Cys255, Tyr473, and Arg643. However, celacinnine did not display any hydrogen bonds. All the hit compounds interacted with all the active site residues except for cystodytin G which did not demonstrate any interaction with the active site residue Ala594. It however displayed interactions with all the other active site residues. These interactions are demonstrated in Supplementary Figure 1.

### 3.3. Molecular Dynamic Simulations for Binding Stability

**Figure 2:**
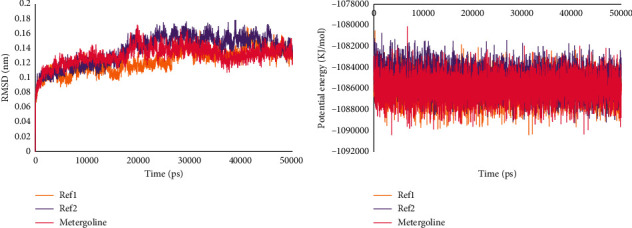
Stability analysis from MD insights. (a) RMSD profiles for POP with Ref1, Ref2, and metergoline; (b) potential energy profiles for POP with Ref1, Ref2, and metergoline.

We performed a 50 ns MD simulation using GROMACS in order to access the binding stability of POP when bound to Ref1, Ref2, and metergoline. RMSD for backbone atoms and potential energy calculations were executed to determine the stability of protein-ligand complex. As illustrated in Figure 2(a), very low average backbone RMSDs of 0.12 nm, 0.13 nm, and 0.13 nm for Ref1, Ref2, and metergoline, respectively, indicated that the protein-ligand complexes were stable. Also, the potential energy profile for all the three systems remained largely unvaried throughout the simulation as depicted in Figure 2(b).

**Figure 3:**
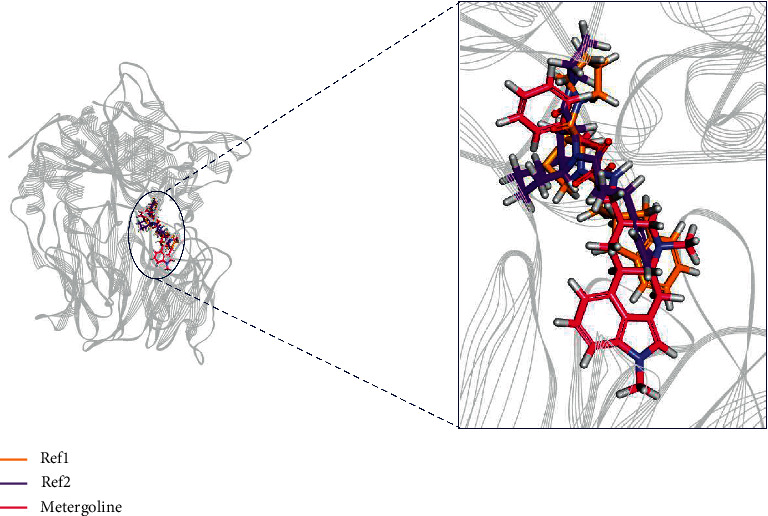
Binding mode analysis of POP with Ref1, Ref2, and metergoline. (a) Superimposed image of representative structures; (b) enlarged view. Protein is shown in grey wire model and compounds are depicted in stick models.

As the RMSD and potential energy profiles were reflecting stability of protein-ligand complexes, reference structures for all the three systems from last 5 ns were extracted and superimposed to check if the binding mode was retained. This analysis, as depicted in Figure 3, revealed that metergoline was retained at the active site while holding onto the binding mode of the reference molecules.

**Figure 4:**
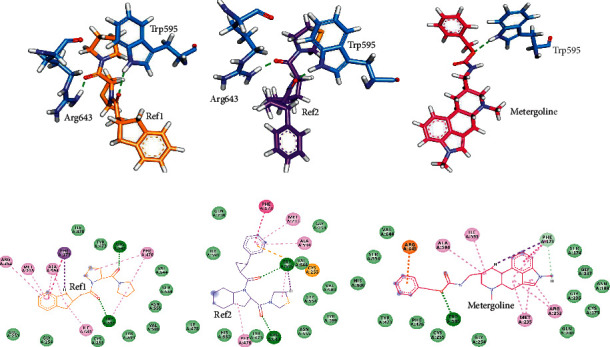
Post-MD intermolecular interactions. Upper panel (a–c) demonstrates hydrogen bond interactions of POP residues (blue) with (a) Ref1, (b) Ref2, and (c) metergoline. Lower panel (d–f) is the 2D representation of all the molecular interactions between POP and (d) Ref1, (e) Ref2, and (f) metergoline. Green dashed lines in upper and lower panel represent hydrogen bonds. All the other dashed lines represent various types of *π* bonds. Light green colored spheres indicate the residues participating in van der Waals interactions.

Further, hydrogen bond interactions sustained for Ref1 and Ref2 with Trp595 and Arg643. In case of metergoline, hydrogen bond with Trp595 was persistent and a new *π* interaction was established with Arg643. Additionally, metergoline also formed a carbon-hydrogen bond with Phe173. Ref1 and Ref2 interacted with all the active site residues. Apart from the interactions with Asn555, Val580, and Tyr599, interactions with all the other active site residues were maintained in case of metergoline. However, metergoline additionally formed van der Waals interactions with other residues.  Figure 4 displays all these interactions.

For JTP-4819 and other hit compounds, we performed 20 ns MD simulation with POP. The average RMSD for pipercallosine, lobeline, martefragin A, lycoperine A, cystodytin G, and celacinnine was observed to be 0.11 nm and for JTP-4819 and hookerianamide J, the average RMSD value was 0.12 nm. The RMSD profiles of all these systems were below the accepted value of 0.15 nm thus indicating stability when bound to POP. Potential energy profiles of these compounds with POP also strengthened and supported the stability observation. The RMSD and potential energy profiles are depicted in Supplementary Figure 2. Owing to the stability conferred by the protein-ligands complexes, the representative structure was picked from the last 5 ns. These representative structures were aligned and superimposed. All the hit molecules remained at the active site. Although martefragin A, lycoperine A, and cystodytin G largely remained at the active site, compared to other molecules, a slight drift was observed. The aligned and superimposed representative structures of JTP-4819 along with other hit compounds are shown in Supplementary Figure 3. Upon checking for the hydrogen bond interactions after MD simulations, it was learnt that JTP-4819 lost hydrogen bond interactions with Arg128 and Arg643. However, hydrogen bond interaction with Cys255 persisted. Interestingly, pipercallosine which had demonstrated only one hydrogen bond interaction with Arg643 prior to MD formed an additional hydrogen bond with Trp595. Hookerianamide J retained its hydrogen bond with Arg643. Although its interaction with Cys255 was lost, hookerianamide J formed a new hydrogen bond interaction with Trp595. Lobeline maintained its hydrogen bond interaction with Arg643. Surprisingly, all the hydrogen bond interactions observed in the docked pose of martefragin A were lost after MD and a new hydrogen bond was formed with Gly254. Cystodytin G and lycoperine A did not demonstrate any hydrogen bond. Similar to the interactions before MD, celacinnine failed to show any hydrogen bond interaction. These hydrogen bonds along with the other *π* and van der Waals interactions formed by the hit compounds with POP are detailed in Supplementary Figure 4.

## 4. Discussion

Due to POP's ability to alter several aspects and function of CNS such as learning, memory, mood, and hypertension [29] and its involvement in alpha synuclein accumulation, POP surfaces out as an appealing target for PD. Although the initial clinical trials results were promising for Z-321, S 17092, and JTP-4819, these were discontinued [12]. JTP-4819 had demonstrated poor BBB permeability [30]. Moreover, safety-related concerns always exist in drug discovery processes. Plants harbor a major reserve of pharmaceutically active compounds and accordingly, interest in natural products based drugs discovery is on the rise. Owing to the therapeutic ability of alkaloids in PD mentioned in the introduction, our work was aimed at exploiting them against POP, computationally. The results demonstrate the POP inhibitory ability of certain alkaloids with metergoline, pipercallosine, hookerianamide J, and lobeline emerging out as notable potential POP inhibitors.

Metergoline is an ergoline alkaloid obtained from taxa such as fungi and some higher plants [31]. There are studies reporting the various therapeutic roles of metergoline: for anti-microbial activity [32, 33] and as antidepressant in seasonal affective disorders [34]. Of interest in relation to Parkinson's disease where the dopamine receptor level is seen to be imbalanced [35], metergoline has been shown to have high affinity for D1 and D4 dopamine receptors [36]. In addition to this, it has been shown that voltage-gated sodium channels have a role in cognitive impairments of PD in rats [37] and Lee and colleagues have recently identified the role of metergoline in inhibiting neural Na^+^ channels [38]. Pipercallosine is an alkaloid enamide isolated from various *Piper* species [39–41]. It is an active apoptosis inducer [41]. Hookerianamide J is a steroid alkaloid obtained from *Sarcococca hookeriana.* It is shown to possess antileishmanial and antibacterial activity and is an effective cholinesterase inhibitor [42]. Lobeline is an optically active piperidine alkaloid obtained from plants particularly in Genus *lobelia.* It binds to and activates a nicotinic acetylcholine receptor and has been shown to be useful in protection of dopaminergic neurons and in reducing the symptoms of parkinsons disease [17, 43].

Upon performing molecular docking of POP with prospective compounds, the GoldScore fitness of Z-321, S-17092, and JTP-4819 was 70.5265, 68.4808, and 60.0431, respectively. Metergoline with a GoldScore fitness of 72.6547 surpassed all the above mentioned inhibitors, while pipercallosine showed a slightly higher GoldScore fitness value of 68.6628 as compared to S-17092. Celacinnine, lobeline, cystodytin G, lycoperine A, hookerianamide J, and martefragin A showed higher GoldScore fitness as compared to JTP-4819 (Supplementary Table 1). Additionally, we also docked berberine against POP which resulted in Goldscore fitness value of 49.319. It was observed that berberine formed interactions with fewer active site residues (Cys255, Phe476, Asn555, Val580, Trp595, Arg643, and Val644) as compared to our hit molecules (data not shown). All our identified alkaloid hits have achieved much higher score than berberine and because berberine has already been tested in vitro for POP inhibitory activity, we believe our compounds will have a greater edge over demonstrating in vitro POP inhibition.

**Figure 5:**
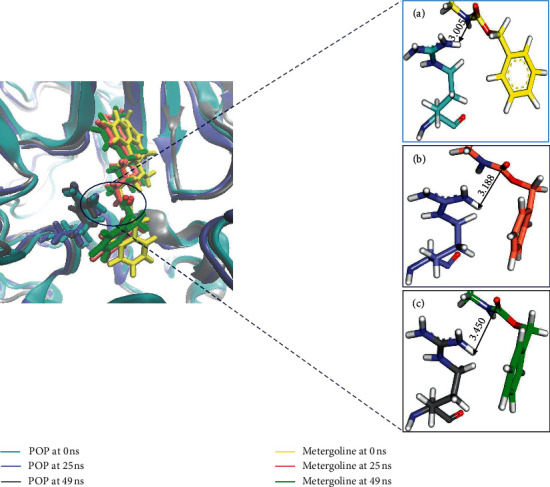
Left image displays superimposed metergoline-POP complex at 0, 25, and 49 ns. Right panel displays the enlarged view of Arg643 and metergoline while highlighting the distance between HH11 of Arg643 with O25 of metergoline at (a) 0 ns, (b) 25 ns, and (c) 49 ns.

Phe476, Asn555, Val580, Trp595, Tyr599, and Val644 form the S1 Subsite of active site, Arg643 forms the S2 subsite and S3 subsite is formed by Phe173, Met235, Cys255, Ile591, and Ala594. The best docked poses of all our hits molecules demonstrated interactions with all these residues, except only for cystodytin G which apart from Ala594 interacted with all the residues. Interaction monitoring after MD analysis of these hit compounds with POP revealed that metergoline, pipercallosine, hookerianamide J, and lobeline demonstrated crucial hydrogen bond interaction with either Trp595 or Arg643 or with both. Pipercallosine and hookerianamide J both showed both Trp595 and Arg643 hydrogen bond interactions. Metergoline and lobeline demonstrated hydrogen bond interaction with Trp595 and Arg643, respectively. All these compounds were retained at the active site as was observed from the aligned and superimposed MD poses (Figure 3 and Supplementary Figure 3). Because metergoline has demonstrated the best molecular docking scores and docked pose had indicated hydrogen bond interactions with Arg643, which was lost after MD, we looked for reasons behind this loss. It was learnt that this hydrogen bond was formed by HH11 (hydrogen) of Arg643 from POP with O25 (oxygen) of metergoline. We compared the metergoline-POP complex profiles at 0, 25, and 49 ns as depicted in Figure 5. Firstly, it was learnt that Arg643 was part of a loop; therefore, it had freedom to move. Secondly, it was observed that the benzene ring in metergoline had moved during the course of simulation. Owing to these reasons, the distance between HH11 of Arg643 and O25 of metergoline had moved from an initial distance of 3.005 Å at 0 ns to 3.188 Å at 25 ns and to 3.450 Å by the end of simulation, thus resulting in loss of the hydrogen bond. However, interaction with Arg643 was pursued in the form of *π* -cation interaction.

GSK552 docking with POP resulted in a GoldScore fitness value of 73.88. Additionally, interaction analysis of docked pose of GSK552 also demonstrated hydrogen bonds with Trp595 and Arg643. We also performed a 20 ns MD for GSK552 in complex with POP. The average RMSD was observed to be 0.09 nm (Supplementary Figure 2). Trp595 and Arg643 hydrogen bonds were preserved and interactions with all the other active site residues were also observed. The 2D representation of the docked pose and MD representative structure of GSK552 are indicated in Supplementary Figures 1 and 4, respectively.

It has been reported that, for a drug to efficiently cross BBB, its molecular weight should be in the range of 314–420 Da, it should be highly lipophilic, i.e., logP should be in the range of 0.66–6, PSA less than 60–70 Å^2^, H-bond donors should be <2, H-bond acceptors should be less than <6, and total number of nitrogen + oxygen atoms should be less than 6 [30, 44].

Literature survey indicated that while Ref1, Ref2 [30], and berberine [45] were capable of crossing BBB, JTP-4819 demonstrated poor BBB permeability [30].  Table 1 compares the properties of Ref1, Ref2, and JTP4819 with metergoline, pipercallosine, hookerianamide J, and lobeline. While Ref1 and Ref2 satisfied the conditions of crossing BBB, with a logP of 0.527, PSA of 89.94, and total number of N + O atoms exceeding 6, JTP-4819 did not satisfy the conditions of BBB permeability, probably which is why it was not able to cross the BBB in clinical trials. Metergoline, pipercallosine, and lobeline satisfied all these conditions of crossing BBB. While hookerianamide J satisfied all the other conditions, its molecular weight is slightly higher (442.677 Da) than the accepted range of efficient BBB crossers, i.e., 314–420 Da. These results further strengthen our proposal for testing them as prospective CNS drugs. Properties pertaining to effective BBB crossing for GSK552, berberine, and other compounds are given in Supplementary Table 2.

Although this research was started keeping Parkinson's disease in mind, literature survey during the process highlighted the role of POP inhibitors not just in PD but in various other disorders such as schizophrenia [30], autism [46], Huntington disease [47], hepatocyte steatosis [48], and acute myeloid leukemia [49]. The role of POP in Alzheimer's disease is contradictory, where certain studies have reported increased POP activity in AD [50] and others have reported decreased POP activity [51, 52]. Based on this, we speculate that the identified hit compounds might be potential pharmaceutical candidates in host of other diseases as well.

## 5. Conclusion

Safety of potential drug candidates is of utmost importance in development of new drugs. Due to their abundance and relative low toxicity, research in identifying plant-based drugs has seen an uptrend. Additionally, crossing of BBB is a major roadblock but an absolute necessary requirement for development of CNS drugs. Using in silico approaches, this work unravels the likely ability of alkaloids in inhibiting prolyl oligopeptidase, which has been indicated as therapeutic target in many diseases. By comparing POP inhibitors that had reached clinical trials, our work displayed better affinity of metergoline, pipercallosine, hookerianamide J, and lobeline for POP. Finally, MD simulations confirmed their stability in complex with POP. Therefore, the research conducted here provides promising results for further investigation of metergoline, pipercallosine, hookerianamide J, and lobeline as effective POP inhibitors.

## Figures and Tables

**Table 1:** Comparison of BBB permeability properties.

Properties	Ref1	Ref2	JTP-4819	Metergoline	Pipercallosine	Hookerianamide J	Lobeline
Molecular weight (Da)	344.5	384.5	359.185	403.517	329.433	442.677	337.455
logP	2.378	3.203	0.527	4.249	4.758	4.935	3.933
Polar surface area (Å^2^)	65.92	65.92	89.94	46.5	47.56	52.57	40.54
Hydrogen bond donor	0	0	2	1	1	2	1
Hydrogen bond acceptor	3	3	4	3	3	3	3
N + O atoms	4	4	7	5	4	4	3

## Data Availability

The datasets generated or analyzed in the current study are available from the corresponding author upon request.

## Supplementary Materials

Supplementary Materials Supplementary Table 1: docking scores of hit compounds and reference inhibitors. Supplementary Figure 1: molecular docking-based intermolecular interactions of JTP-4819, GSK552, and other hit compounds. Supplementary Figure 2: RMSD and potential energy profiles of JTP-4819, GSK552, and other hit compounds. Supplementary Figure 3: binding mode analysis of POP with JTP-4819, GSK552, and other hit compounds. Supplementary Figure 4: post-MD intermolecular interactions of JTP-4819, GSK552, and other hit compounds. Supplementary Table 2: BBB permeability properties of celacinnine, cystodytin G, lycoperine A, martefragin A, berberine, and GSK552. Supplementary Figure 5: chemical structures of the proposed inhibitors.
