# Supplementary material for: Computer-Aided Drug Discovery Identifies Alkaloid Inhibitors of Parkinson's Disease Associated Protein, Prolyl Oligopeptidase
Source: Evid Based Complement Alternat Med. 2021 Apr 8;2021:6687572. doi: 10.1155/2021/6687572 (PMC8052153; doi:10.1155/2021/6687572)

| **Chemical Name** | **GoldScore Fitness** |
| --- | --- |
| Metergoline | 72.6547 |
| Ref1 | 70.5265 |
| Pipercallosine | 68.6628 |
| Ref2 | 68.4808 |
| Celacinnine | 66.0825 |
| Lobeline | 65.0957 |
| Cystodytin G | 62.6932 |
| Lycoperine A | 61.4279 |
| Hookerianamide J | 61.3176 |
| Martefragin A | 60.5302 |
| JTP-4819 | 60.0431 |

**Supplementary table1:** Docking scores of hit compounds and reference inhibitors

**Supplementary figure1.** Molecular docking based intermolecular interactions. 2D representation of all the molecular interactions between POP and **A)** JTP-4819, **B)** GSK552 and **C)** Pipercallosine **D)** Hookerianamide J **E)** Lobeline **F)** Martefragin A **G)** Lycoperine A **H)** Cystodytin G and **I)** Celacinnine . Green dashed lines represent hydrogen bond. Blue dashed line represents a Halogen bond. All the other dashed lines represent various types of π bonds. Light green colored spheres indicate the residues participating in van der Waals interactions


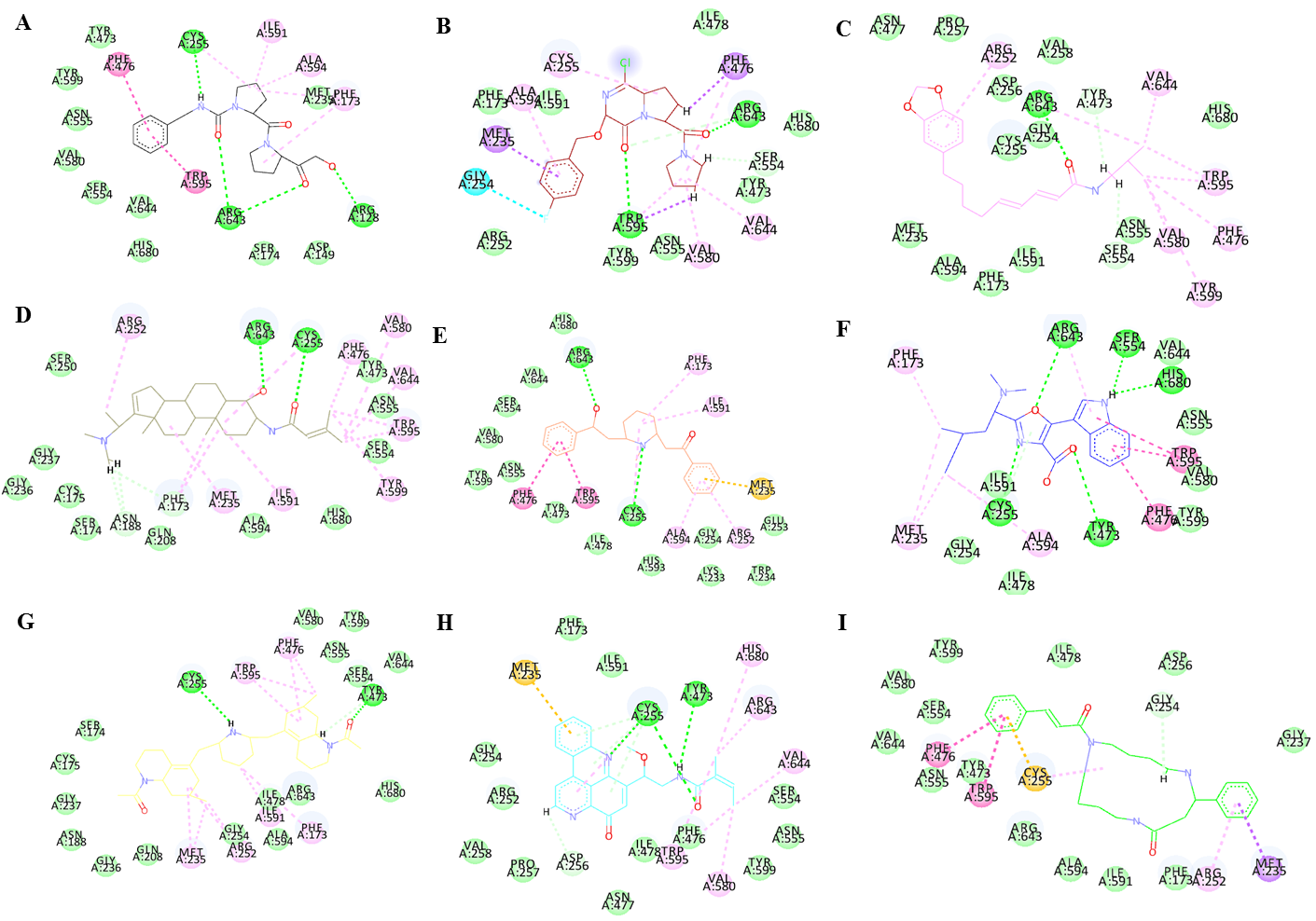


**Supplementary figure2.** Stability analysis from MD insights. **A)** RMSD profiles for POP with JTP5819, GSK552 and other hit compounds; **B)** Potential energy profiles for POP with JTP5819, GSK552 and other hit compounds

**
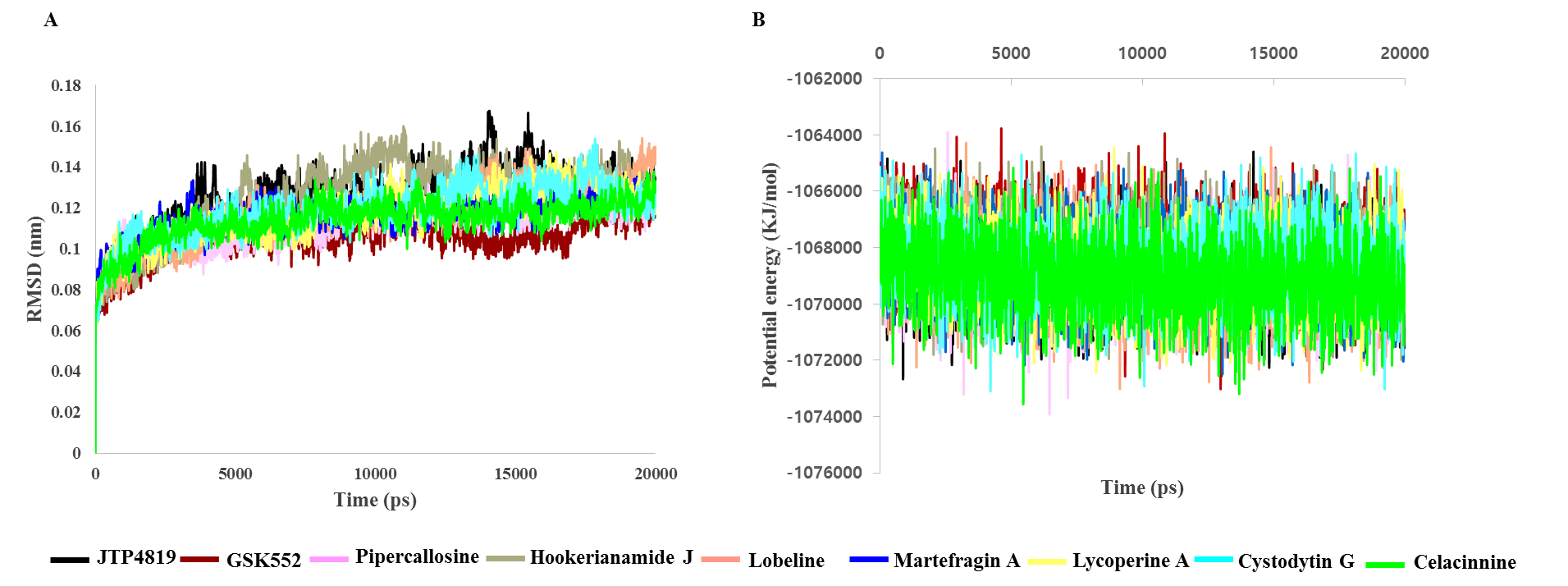
**

**Supplementary figure3**: Binding mode analysis of POP with JTP-4819, GSK552 and other hit compounds **A)** Superimposed image of representative structures; **B)** enlarged view. Protein is shown in grey wire model and compounds are depicted in stick models

**
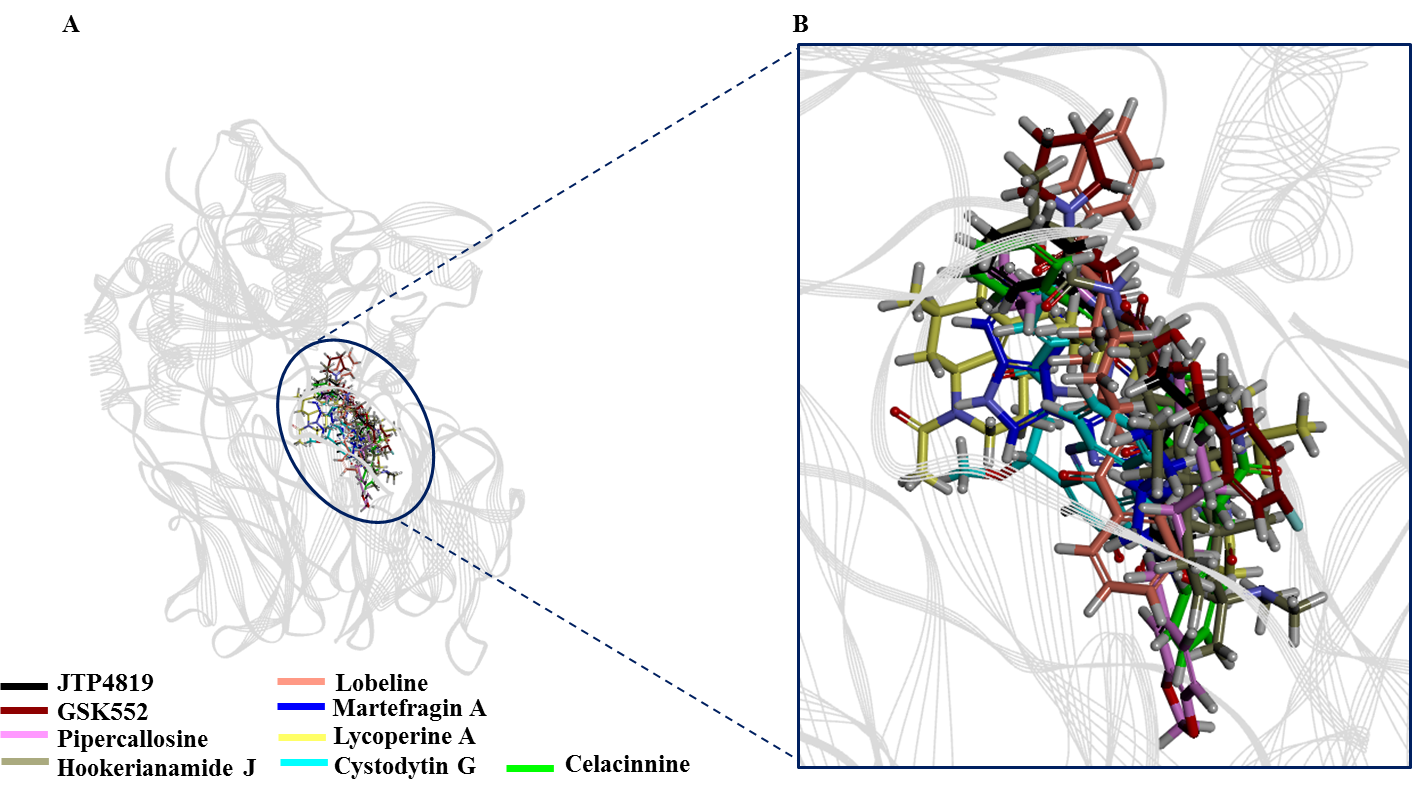
**

**Supplementary figure4.** Post MD intermolecular interactions. 2D representation of all the molecular interactions between POP and **A)** JTP-4819, **B)** GSK552 and **C)** Pipercallosine **D)** Hookerianamide J **E)** lobeline **F)** Martefragin A **G)** Lycoperine A **H)** Cystodytin G and **I)** Celacinnine . Green dashed lines represent hydrogen bond. All the other dashed lines represent various types of π bonds. Light green colored spheres indicate the residues participating in van der Waals interactions

**
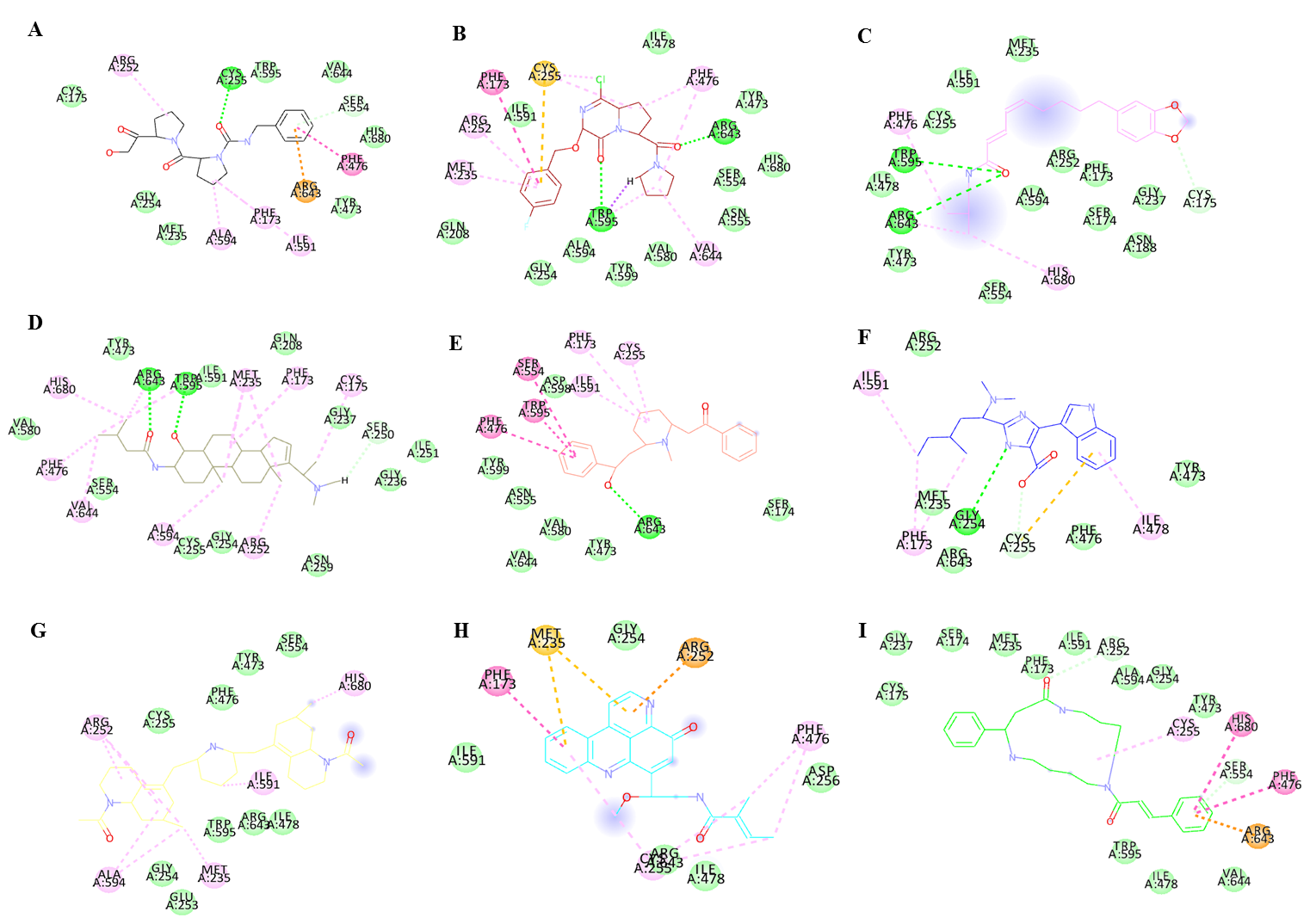
**

**Supplementary table2**: BBB permeability properties of celacinnine, cystodytin G, lycoperine A, martefragin A, berberine and GSK552

| **Properties** | **Celacinnine** | **Cystodytin G** | **Lycoperine A** | **Martefragin A** | **Berberine** | **GSK552** |
| --- | --- | --- | --- | --- | --- | --- |
| **Molecular weight(Da)** | 405.533 | 387.431 | 495.74 | 355.431 | 336.361 | 393.84 |
| **logP** | 2.758 | 3.204 | 4.571 | 1.293 | 3.962 | 2.119 |
| **Polar Surface Area (Å^2^ )** | 61.44 | 81.18 | 52.65 | 82.36 | 40.8 | 62.2 |
| **Hydrogen bond donor** | 2 | 1 | 1 | 2 | 0 | 0 |
| **Hydrogen bond acceptor** | 3 | 5 | 3 | 4 | 4 | 4 |
| **N+O atoms** | 5 | 6 | 5 | 6 | 5 | 6 |

**Supplementary figure5:** Chemical structures of proposed inhibitors


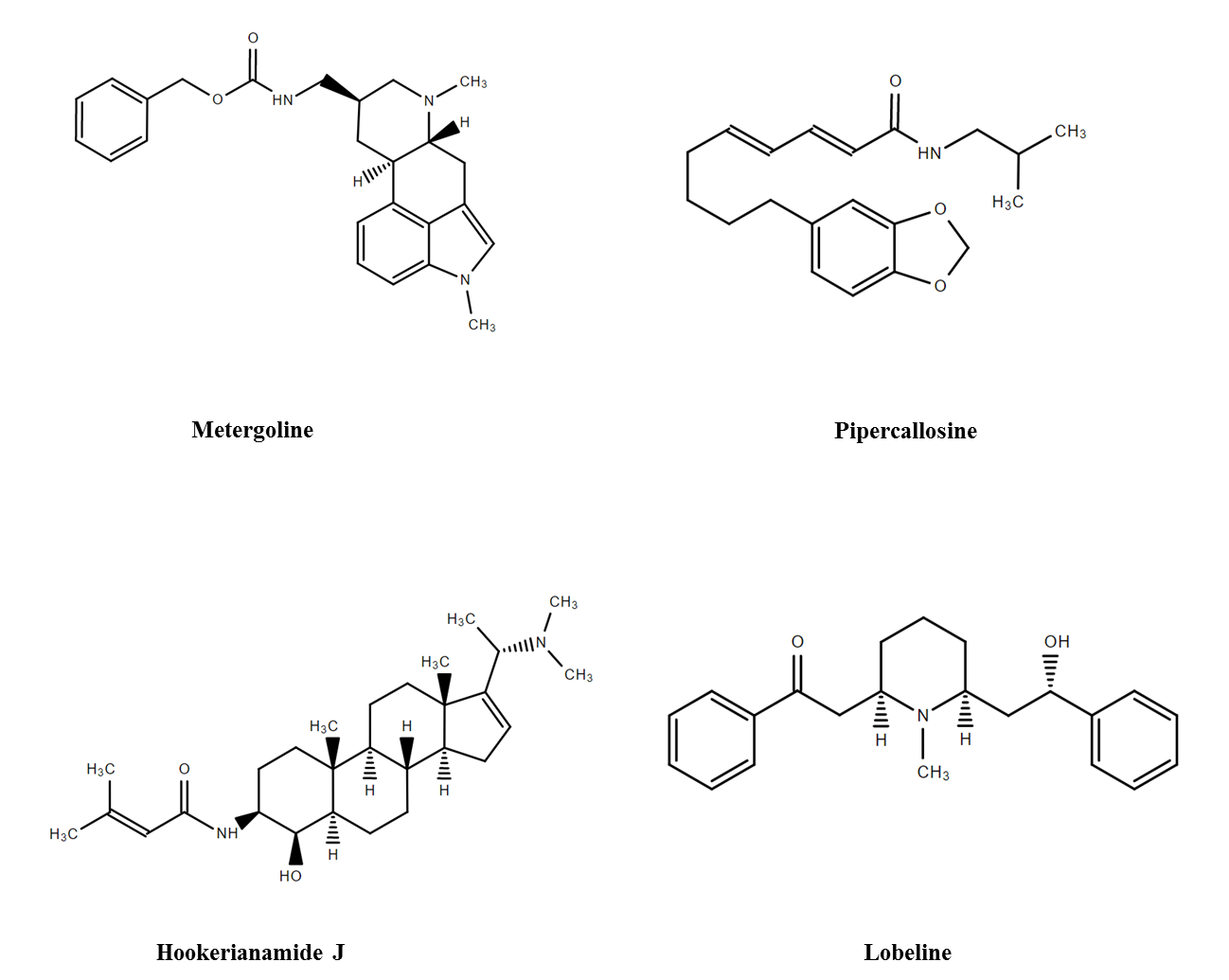

Supplement: Supplementary Materials — Supplementary Table 1: docking scores of hit compounds and reference inhibitors. Supplementary Figure 1: molecular docking-based intermolecular interactions of JTP-4819, GSK552, and other hit compounds. Supplementary Figure 2: RMSD and potential energy profiles of JTP-4819, GSK552, and other hit compounds. Supplementary Figure 3: binding mode analysis of POP with JTP-4819, GSK552, and other hit compounds. Supplementary Figure 4: post-MD intermolecular interactions of JTP-4819, GSK552, and other hit compounds. Supplementary Table 2: BBB permeability properties of celacinnine, cystodytin G, lycoperine A, martefragin A, berberine, and GSK552. Supplementary Figure 5: chemical structures of the proposed inhibitors. [file 6687572.f1.docx]
